# Lower reoperation rate for cemented femoral stem than for uncemented femoral stem in primary total hip arthroplasty following a displaced femoral neck fracture

**DOI:** 10.1051/sicotj/2015028

**Published:** 2015-10-16

**Authors:** Michelle F. Andersen, Thomas Jakobsen, Anne S. Bensen, Niels Krarup

**Affiliations:** 1 Department of Orthopaedic Surgery, The Regional Hospital in Viborg Heibergs Allé 4 8800 Viborg Denmark; 2 Orthopaedic Research Unit, Aarhus University Hospital Tage-Hansens Gade 2 8000 Aarhus C Denmark; 3 Department of Orthopaedic Surgery, Odense University Hospital Sdr. Boulevard 29 5000 Odense C Denmark

**Keywords:** Displaced femoral neck fracture, Total hip arthroplasty, Cemented, Uncemented, Reoperation

## Abstract

*Introduction*: Acute displaced femoral neck fractures are often treated with cemented hemiarthroplasty (HA). There is increasing evidence that total hip arthroplasty (THA) may be a better alternative, but the degree to which the fixation of the femoral stem used affects the outcome is not fully known. The aim of this study is to compare rates of operative complications and implant survival following THA treatment of displaced femoral neck fractures with either a cemented or an uncemented femoral stem.

*Methods*: The study consists of two groups of patients (*N* = 334), who were treated for a displaced femoral neck fracture with THA at the Regional Hospital of Viborg during 2007–2012. The first group (50.9%) had uncemented (*Corail*^*®*^) stem while the second group (49.1%) had cemented (*Exeter*^*®*^) stem implanted. Nearly all patients had uncemented dual mobility cup (*Saturne*^*®*^) as acetabular component and were followed up to three months postoperatively. Data regarding rates of implant survival and operative complications were obtained by retrospective review of medical records.

*Results*: We found a statistically significant difference regarding rates of postoperative reoperation with 1.2% (95% CI 0.005–0.03) for cemented and 5.9% (95% CI 0.02–0.09) for uncemented stem (*p* = 0.02). The main causes for reoperation were peri-prosthetic fractures and deep infections. There was no difference regarding dislocation or peroperative complications. Rates of dislocation were 4.3% (95% CI 0.012–0.07) for cemented and 3.5% (95% CI 0.008–0.06) for uncemented stem (*p* = 0.72). Rates of peroperative complications were 6.1% (95% CI 0.024–0.1) for cemented and 8.2% (95% CI 0.04–0.12) for uncemented stem (*p* = 0.1).

*Discussion*: Our results indicate that cemented femoral stem is superior to cementless when rates of reoperation are compared.

## Introduction

A femoral neck fracture is a common injury and the incidence is high in the elderly population with an estimated 1.26 million hip fractures in 1990. In developed countries the incidence is expected to further increase to between 7.3 and 21.3 million hip fractures by the year 2050 due to continued ageing of the general population as well as increased life expectancy and will therefore become a major challenge for the health-care system over the next decades [1].

Hemiarthroplasty (HA) has traditionally been the preferred primary treatment for acute displaced fractures of the femoral neck (Garden stage III and IV [2]), but there is increasing evidence that total hip arthroplasty (THA) may be a better alternative [3–6]. However, because of this long going tradition the majority of the studies published regarding whether the use of cement or not has been made with HA stems – a debate which is still controversial despite continuous research [7–9]. For THA stem, very little has been published on the subject [10].

The femoral component in hip arthroplasty can be implanted with or without the use of cement. A systematic Cochrane review from 2010 on arthroplasties for proximal femoral fractures [3] reported that cementing the femoral stem may provide a more stable fixation and can reduce postoperative thigh pain as well as lead to better mobility compared to the cementless design. Fewer implant-related complications and reduced reoperation rates, especially postoperative peri-prosthetic femoral fractures, have also been reported [3, 8–11]. However, the use of cement also has disadvantages as it may trigger a rare but severe risk of cardiovascular complications due to embolism [12, 13], the length of surgery is longer and the blood loss greater compared to the use of uncemented prosthesis [14, 15]. No difference in mortality between the two methods has been reported [3, 14, 16].

The aim of the present study was to compare rates of implant survival and operative complications within three months following primary THA treatment of displaced femoral neck fractures with either a cemented or an uncemented femoral stem including a dual mobility cup as acetabular component. We hypothesized that the use of a cemented femoral component would be associated with a decreased risk of postoperative complications compared with the uncemented stem, especially regarding reoperations.

## Materials and methods

We retrospectively identified and reviewed all patients who underwent primary surgery for a displaced femoral neck fracture and were treated with either a cemented or an uncemented THA femoral stem at the Regional Hospital of Viborg in Denmark between 1 January 2007 and 31 December 2013.

During the period 2007–2011, the standard treatment for primary THA in our department was a cementless femoral stem (*Corail*^®^, DePuy Orthopaedics, Warsaw, IN, USA). However, due to the good results shown in the literature regarding postoperative complications when using cemented HA stem, it was decided to change the treatment to cemented THA femoral stem (*Exeter*^®^, Stryker Orthopaedics, Mahwah, NJ, USA) in January 2012. During the whole period, the THA was primarily made with an uncemented dual mobility cup, DMC (*Saturne*^*®*^, Amplitude, Neyron, France), as acetabular component to decrease dislocation rate [4].

The patients were obtained by retrospective review of the departments’ medical records, including anaesthetic data. A total of 334 consecutive cases (mean age, 77.8 years) with either a cementless (*N* = 170) or a cemented (*N* = 164) THA were retrospectively evaluated. Patients with pathological fractures and implanted femoral stem other than *Corail*^*®*^ or *Exeter*^®^ were not included.

For all patients, data was retrospectively collected in the following categories as primary outcome measures; peroperative complications, dislocation and reoperation (defined as an operation of any kind after primary surgery, exclusive of closed reduction of the dislocated arthroplasties). As secondary outcome measures, we obtained information on waiting time from hospitalization to surgery, duration of surgery (incision to close), perioperative blood loss, surgeon’s experience and mortality. All patients were followed until the time of dislocation, reoperation, death or emigration up to 3 months after primary surgery.

The National Registry of Patients (NRP) is a national-based patient administration system in Denmark, which contains data on all admissions and discharges from hospitals in Denmark, including surgical procedures as well as information about reoperations. We have cross-checked all our data with data from NRP to ensure the inclusion of all patients treated for dislocation and reoperation in other hospitals in Denmark during the follow-up period. None of the included patients had been treated in other hospitals than the Regional Hospital of Viborg after primary surgery. Data on mortality and emigration were retrieved from The Central Office of Civil Registration. Approval from The Danish Data Protection Agency, and The Danish Health and Medicines Authority was obtained before initiation of the study.

All patients received complete medical evaluation and clearance for anaesthesia before surgery; spinal analgesia was used in the majority of the patients.

The department used the standard Hvidovre algorithm [17] for surgical treatment regarding the choice of implant. The only exception to the algorithm was using THA femoral stem with a dual mobility cup instead of HA. In each case, the THA was implanted via posterior approach including reconstruction of the posterior capsule and reinsertion of the external rotators during closing. In the cemented group, the stem was fixated by using Rifobacin^®^ Bone Cement R (Biomet) containing the antibiotic Gentamycin. It was mixed in a closed system named Optipac.

All patients were administered preoperatively intravenous Cefuroxim 1.5 g single-dose as well as 1.5 g three times a day, the first day after surgery as prophylactic antibiotic. A postoperative X-ray was taken the day after surgery. After the operation, all patients underwent the same rehabilitation programme instructed by the hospital physiotherapists, which included early mobilization and full weight-bearing training within threshold of pain. They were informed to avoid hip flexion above 90°, and internal rotation and adduction above 0° for three months. All patients were followed, up to three (0–90 days) months postoperatively.

## Statistics

Results are reported as means with standard deviations (SD) or frequencies with 95% confidence intervals (95% CI). When appropriate, ranges are supplied. Statistical analysis was performed using STATA statistical software (version 9.0; Stata Inc., College Station, TX, USA) and the level of significance was defined as a *p*-value of <0.05. Categorical data were analysed using the chi-square test. For continuous data, a Student’s *t*-test was used.

## Results

The results for primary and secondary parameters are shown in Table 1.

**Table 1.** Results.

Parameter	Cemented THA stem	95% CI	*SD*	Uncemented THA stem	95% CI	*SD*	*p* value
Peroperative complications	10/164 (6.1%)	0.024–0.10%	–	14/170 (8.2%)	0.04–0.12%	–	0.1
Dislocation	7/164 (4.3%)	0.012–0.07%	–	6/170 (3.5%)	0.008–0.06%	–	0.72
Reoperation	2/164 (1.2%)	0.005–0.03%	–	10/170 (5.9%)	0.02–0.09%	–	0.02
Waiting time from hospitalization to surgery, mean (h)	30	27–32	19.0	25	21–29	16	0.06
Duration of surgery, mean (min)	84	78–89	24.3	74	70–79	28.2	0.0099
Perioperative blood loss, mean (mL)	392	345–439	269.0	378	343–410	213	0.6
Three-month mortality postoperatively	27/164 (16.5%)	0.10–0.21%	–	15/170 (8.8%)	0.05–0.14%	–	0.06

We found a statistically significant difference regarding rates of postoperative reoperations of any kind, in favour of cemented THA femoral stem (*p* = 0.02). Reoperations were required in two patients (1.2%) (95% CI 0.005–0.03%) treated with a cemented stem and 10 patients (5.9%) (95% CI 0.02–0.09%) treated with the uncemented stem. The different causes for reoperations are listed in Table 2. The most frequent reoperation was a soft-tissue procedure for infection, one superficial in the cemented group and four deep infections in the cementless group. Four patients split between the two groups had experienced a traumatic peri-prosthetic fracture that needed surgery.

**Table 2.** Causes for reoperation.

Causes for reoperation	Cemented THA stem (*n*)	Uncemented THA stem (*n*)
Postoperative peri-prosthetic fracture	1	3
Aseptic loosening	0	2
Haematoma formation postoperatively	0	1
Deep infection	0	4
Superficial infection	1	0

The occurrence of both peroperative complications (all small fissures and minor fractures) and dislocations were similar in the two groups with no statistical significance found. All the dislocations for both groups happened within the first 50 days after primary surgery, and the iatrogenic fractures were all reparable with cerclage wiring alone or with a trochanteric grip under the primary surgery. All instances healed well during the follow-up.

The cohort had a statistically significant difference regarding duration of surgery (*p* = 0.0099) in favour of uncemented THA (74 min vs. 84 min). There was no statistical difference in waiting time from hospitalization to surgery or perioperative blood loss between the two groups of patients.

During the three months follow-up, there was a notable trend of lower mortality in patients who received a cementless THA (8.8%) compared to the cemented group (16.5%), but it did not reach statistical significance (*p* = 0.06). No patients died peroperatively but there were three postoperative deaths within the first 72 hours in the cemented group compared to none in the uncemented, two suspected as cardiovascular disturbance and one as renal failure.

We found no relation between the surgeon’s level of experience and the number of peroperative complications, dislocations or reoperations in both groups. These were evenly divided between junior surgeons alone, junior surgeons supervised by senior surgeons and senior surgeons compared to the number of operations made. Senior surgeons mainly performed the operations in both groups (42.7% cemented vs. 52.9% uncemented).

The baseline characteristics of the population are shown in Table 3. The two groups are comparable to each other except for the age (*p* < 0.00), where the patients treated with a cemented THA tended to be older with an average age of 80.9 years (*SD* = 9.2, range 53–101 years) compared to 74.8 years (*SD* = 11.40, range 43–98 years) in the uncemented group. The mean follow-up time was 77.5 days for cemented THA and 80.1 for uncemented.

**Table 3.** Population characteristics.

Variable	Cemented THA stem (*N* = 164)	Uncemented THA stem (*N* = 170)
Sex, *n*, male/female (%)	49 (29.9%)/115 (70.1%)	52 (30.6%)/118 (69.4%)
Age at fracture*, mean, years (range)	80.9 (53–101)	74.8 (43–98)
Femoral stem, type	Exeter^®^	Corail^®^
Femoral stem, type, with/without cement (%)	164/164 (100%)	170/170 (100%)
Dual mobility cup, type	Saturne^®^	Saturne^®^
Dual mobility cup, *n*, with/without cement (%)	6/159 (3.8%)	1/169 (0.6%)
Surgical approach	Posterolateral	Posterolateral
Follow-up, mean (days)	77.5	80.1

* *p* < 0.00.

## Discussion

The purpose of this study was to compare rates of operative complications and implant survival following primary THA treatment of displaced femoral neck fractures with either a cemented or an uncemented femoral stem. Our results show that cemented THA femoral stem reduces the reoperation rate compared to cementless stem.

By changing our regimen of treatment from cementless stem (*Corail*^*®*^) to cemented (*Exeter*^®^), we reduced the number of reoperations in our population from 5.9% to 1.2%. This finding is very similar to those of the few published studies that are comparing whether to use cemented or cementless THA femoral stem after acute displaced femoral neck fractures. A recent study made of results from the National Joint Registry of England and Wales [10] showed a revision rate after five years of 4.1% in the group not using cemented components compared to 0.9% in the group who used cement. Several studies and systematic reviews have been performed to clarify this issue for HA and the tendency remains the same, although not quite as pronounced [3, 8–11].

The more forceful procedure of trying to achieve a tight interference fit of the uncemented prosthesis compared to cemented devices where a space is left for the cement to act as a grout, makes iatrogenic fissures and minor fractures in the femur during implantation a severe risk with the cementless prosthesis [18]. As the surgeon often does not recognize these complications during surgery, it may result in an occult femoral fracture that is first diagnosed days or even months after surgery. This may explain our high reoperation rate due to postoperative peri-prosthetic fractures in the cementless group, as all our patients have osteoporotic bone. Comparing our results with reported rates of reoperation in other studies for HA, our reoperation rate for peri-prosthetic fracture in THA with cemented stem is similar to the literature [8–11].

Peri-prosthetic fractures as well as deep infections were the most frequent causes for reoperation in the cementless group in our study. There was only one reoperation for superficial infection, this being in the cemented group. The prophylactic use of antibiotic-loaded bone cement has shown to protect against infection in several studies [19] and it is most likely because it is preventing the bacteria from forming a biofilm on the implant [20]. One explanation for the strong trend towards more infections after uncemented THA could be the absence of cement and thereby no antibiotic bone prophylaxis. Additionally, there were two cases of aseptic loosening in the cementless group compared to no cases in the cemented. This notable discrepancy could be explained by technical errors or minor fractures occurring within the cementless surgery, which could have led to the loosening.

The rate of dislocation is not influenced by the fixation of the femoral stem, but rather by the type of cup being used as well as the placement of the components. Since the same type of cup was used in both groups, an uncemented DMC, it was not surprising to see that the rates of dislocation were the same when comparing the two groups. A find in agreement with a previously published report from our institute using THA with the same uncemented DMC as treatment for displaced femoral neck fractures [4].

Insertion of a cemented prosthesis is a more time demanding procedure compared to the uncemented, as the bone cement has to harden for at least 12 minutes after application before the operation can proceed. Therefore, it is of no surprise that we found that the length of surgery was significantly longer for THA with a cemented femoral stem than for the uncemented procedure.

Our finding favours the cementless design, which is also supported by other studies [3, 14] as well as a randomized controlled trial (RCT) comparing cemented HA with an uncemented HA [9]. Here the duration of surgery was found to be 82.6 min for the cemented design, compared to 70.2 min for the group who received a HA without cement. A difference very similar to our finding, a 10 minute difference.

Cement insertion can trigger a minor but well-documented effect on the cardiopulmonary system resulting in cardiovascular disturbances to sudden death. However, the changes appear to be transient and with no clinical significance [12, 13]. Although we could not demonstrate a statistically significant difference between the mortality of the two groups (*p* = 0.06), there was a notable trend towards the cemented group; the mortality was almost double compared to the patients receiving an uncemented femoral stem (16.5% vs. 8.8%). This result stands in contrast to previous studies made on HA femoral stem, which found no difference in either three-month or five-year mortality [3, 14, 16]. We suggest that the main reasons for our finding are that our cohort is not comparable according to age as well as the patients’ physiological and mental health has not been registered. It is important to emphasize that our study does not have the statistical power to evaluate the potential adverse health effects of cement. However, the lack of any significant difference in mortality reported in the latest Cochrane report on arthroplasties for proximal femoral fractures suggests that the risk of cementing is minimal [3].

Our study is retrospective and thereby limited by the limitations related to the design. The main weakness is the minor cohorts included in the study. It is therefore important to be precautious about the interpretation of our findings of non-significant differences due to the risk of making a type 2 error. However, all patients were operated at the same hospital and since the main source of data was medical records from our hospital, we have been able to get access to most data on our cohorts. In order to optimize completeness of the data, all patients have been cross-checked with the Danish National Patients Register. Another limitation is the lack of registration of the patients’ physiological and mental health in both groups. This may have an adverse influence on the patients’ compliance during the follow-up period. Furthermore, our cohorts are not comparable according to age; there is a significant difference in mean age by six years between the two groups. We have no explanation for this difference, as the department used the same surgery strategy regarding the choice of implant for all patients included by using the Hvidovre algorithm.

Based on the presented results and the good evidence on using cement for primary HA in the existing literature, the department is continuing to use cement for primary THA after acute displaced femoral neck fractures.

## Conclusion

In conclusion, our results suggest that cemented femoral stem in primary THA after acute displaced femoral neck fractures is superior to cementless when rates of reoperation are compared. However, further RCTs with focus on both early operative complications and implant survival are necessary to support the evidence and determine the optimal treatment for displaced femoral neck fractures.

## Conflict of interest

The authors declare that they have no conflict of interest.

## References

[1] Gullberg B, Johnell O, Kanis JA (1997) International original article world-wide projections for hip fracture. Osteoporos Int 7(44), 407–413. 9425497 10.1007/pl00004148

[2] Frandsen PA, Andersen E, Madsen F, Skjodt T (1988) Garden’s classification of femoral neck fractures. An assessment of inter-observer. Bone Joint J 70(4), 588–590. 10.1302/0301-620X.70B4.3403602 3403602

[3] Parker MJ, Gurusamy KS, Azegami S (2010) Arthroplasties (with and without bone cement) for proximal femoral fractures in adults. Cochrane Dtabase Syst Rev 16(6). 10.1002/14651858.CD001706.pub4 PMC12360749 20556753

[4] Bensen AS, Jakobsen T, Krarup N (2014) Dual mobility cup reduces dislocation and re-operation when used to treat displaced femoral neck fractures. Int Orthop 38, 1241–1245. 24441666 10.1007/s00264-013-2276-8 PMC4037505

[5] Burgers PTPW, Van Geene AR, Van den Bekerom MPJ, Van Lieshout EMM, Blom B, Aleem IS, Bhandari M, Poolman RW (2012) Total hip arthroplasty versus hemiarthroplasty for displaced femoral neck fractures in the healthy elderly: a meta-analysis and systematic review of randomized trials. Int Orthop 36(8), 1549–1560. 22623062 10.1007/s00264-012-1569-7 PMC3535035

[6] Blomfeldt R, Törnkvist H, Eriksson K, Söderqvist A, Ponzer S, Tidermark J (2007) A randomised controlled trial comparing bipolar hemiarthroplasty with total hip replacement for displaced intracapsular fractures of the femoral neck in elderly patients. J Bone Joint Surg Br 89(2), 160–165. 17322427 10.1302/0301-620X.89B2.18576

[7] Ning G-Z, Li Y-L, Wu Q, Feng S-Q, Li Y, Wu Q-L (2014) Cemented versus uncemented hemiarthroplasty for displaced femoral neck fractures: an updated meta-analysis. Eur J Orthop Surg Traumatol 24(7), 7–14. 23412274 10.1007/s00590-012-1151-4

[8] Khan RJK, MacDowell A, Crossman P, Keene GS (2002) Cemented or uncemented hemiarthroplasty for displaced intracapsular fractures of the hip – a systematic review. Injury 33(1), 13–17. 11879826 10.1016/s0020-1383(01)00101-2

[9] Figved W, Opland V, Frihagen F, Jervidalo T, Madsen JE, Nordsletten L (2009) Cemented versus uncemented hemiarthroplasty for displaced femoral neck fractures. Clin Orthop Relat Res 467(9), 2426–2435. 19130162 10.1007/s11999-008-0672-y PMC2866935

[10] Stafford GH, Charman SC, Borroff MJ, Newell C, Tucker JK (2012) Total hip replacement for the treatment of acute femoral neck fractures: results from the National Joint Registry of England and Wales at 3–5 years after surgery. Ann R Coll Surg Engl 94, 193–198. 22507726 10.1308/003588412X13171221589720 PMC3705235

[11] Gjertsen J-E, Lie SA, Vinje T, Engesæter LB, Hallan G, Matre K, Furnes O (2012) More re-operations after uncemented than cemented hemiarthroplasty used in the treatment of displaced fractures of the femoral neck: an observational study of 11,116 hemiarthroplasties from a national register. J Bone Joint Surg Br 94(8), 1113–1119. 22844055 10.1302/0301-620X.94B8.29155

[12] Viberg B, Overgaard S, Lauritsen J, Ovesen O (2013) Lower reoperation rate for cemented hemiarthroplasty than for uncemented hemiarthroplasty and internal fixation following femoral neck fracture: 12- to 19-year follow-up of patients aged 75 years or more. Acta Orthop 84(3), 254–259. 23594248 10.3109/17453674.2013.792033 PMC3715822

[13] Yli-kyyny T, Sund R, Venesmaa P (2014) Cemented or uncemented hemiarthroplasty for the treatment of femoral neck fractures? A Finnish database study of 25,174 patients. Acta Orthop Scand 85(1), 49–53. 10.3109/17453674.2013.878827 PMC3940991 24397746

[14] Taylor F, Wright M, Zhu M (2012) Hemiarthroplasty of the hip with and without cement: a randomized clinical trial. J Bone Joint Surg Am 94(7), 577–583. 22488613 10.2106/JBJS.K.00006

[15] Donaldson AJ, Thomson HE, Harper NJ, Kenny NW (2009) Bone cement implantation syndrome. Br J Anaesth 102(1), 12–22. 19059919 10.1093/bja/aen328

[16] Yli-Kyyny T, Ojanperä J, Venesmaa P, Kettunen J, Miettinen H, Salo J, Kröger H (2013) Perioperative complications after cemented or uncemented hemiarthroplasty in hip fracture patients. Scand J Surg 102(2), 124–128. 23820689 10.1177/1457496913482249

[17] Palm H, Krasheninnikoff M, Holck K, Lemser T, Foss NB, Jacobsen S, Kehlet H, Gebuhr P (2012) A new algorithm for hip fracture surgery. Acta Orthop 83(1), 26–30. 22248165 10.3109/17453674.2011.652887 PMC3278653

[18] Foster AP, Thompson NW, Wong J, Charlwood P (2005) Periprosthetic femoral fractures – a comparison between cemented and uncemented hemiarthroplasties. Injury 36(3), 424–429. 15710161 10.1016/j.injury.2004.07.023

[19] Engesæter LB, Lie SA, Espehaug B, Furnes O, Vollset SE, Havelin LI (2003) Antibiotic prophylaxis in total hip arthroplasty. Effects of antibiotic prophylaxis systemically and in bone cement on years in the Norwegian Arthroplasty Register. Acta Orthop Scand 74(6), 644–651. 14763692 10.1080/00016470310018135

[20] Gillespie WJ, Walenkamp GHIM (2010) Antibiotic prophylaxis for surgery for proximal femoral and other closed long bone fractures (Review). Cochrane Database Syst Rev 17(3). 10.1002/14651858.CD000244.pub2 PMC7043359 20238310

